# Distribution of chimeric antigen receptor-modified T cells against CD19 in B-cell malignancies

**DOI:** 10.1186/s12885-021-07934-1

**Published:** 2021-02-25

**Authors:** Zhitao Ying, Ting He, Xiaopei Wang, Wen Zheng, Ningjing Lin, Meifeng Tu, Yan Xie, Lingyan Ping, Chen Zhang, Weiping Liu, Lijuan Deng, Meng Wu, Feier Feng, Xin Leng, Tingting Du, Feifei Qi, Xuelian Hu, Yanping Ding, Xin-an Lu, Yuqin Song, Jun Zhu

**Affiliations:** 1 Department of Lymphoma, Key Laboratory of Carcinogenesis and Translational Research (Ministry of Education/Beijing), Peking University Cancer Hospital & Institute, Beijing, China; 2 Beijing Immunochina Pharmaceuticals Co., Ltd., Beijing, China

**Keywords:** CAR-T, Biodistribution, B-ALL, B-NHL, Blood

## Abstract

**Background:**

The unprecedented efficacy of chimeric antigen receptor T (CAR-T) cell immunotherapy of CD19^+^ B-cell malignancies has opened a new and useful way for the treatment of malignant tumors. Nonetheless, there are still formidable challenges in the field of CAR-T cell therapy, such as the biodistribution of CAR-T cells in vivo.

**Methods:**

NALM-6, a human B-cell acute lymphoblastic leukemia (B-ALL) cell line, was used as target cells. CAR-T cells were injected into a mice model with or without target cells. Then we measured the distribution of CAR-T cells in mice. In addition, an exploratory clinical trial was conducted in 13 r/r B-cell non-Hodgkin lymphoma (B-NHL) patients, who received CAR-T cell infusion. The dynamic changes in patient blood parameters over time after infusion were detected by qPCR and flow cytometry.

**Results:**

CAR-T cells still proliferated over time after being infused into the mice without target cells within 2 weeks. However, CAR-T cells did not increase significantly in the presence of target cells within 2 weeks after infusion, but expanded at week 6. In the clinical trial, we found that CAR-T cells peaked at 7–21 days after infusion and lasted for 420 days in peripheral blood of patients. Simultaneously, mild side effects were observed, which could be effectively controlled within 2 months in these patients.

**Conclusions:**

CAR-T cells can expand themselves with or without target cells in mice, and persist for a long time in NHL patients without serious side effects.

**Trial registration:**

The registration date of the clinical trial is May 17, 2018 and the trial registration numbers is NCT03528421.

**Supplementary Information:**

The online version contains supplementary material available at 10.1186/s12885-021-07934-1.

## Background

Chimeric antigen receptor T (CAR-T) cell therapy is drawing more and more attention for treating relapsed or refractory (r/r) B-cell malignancies, including B-cell acute lymphoblastic leukemia (B-ALL) and B-cell non-Hodgkin lymphoma (B-NHL) [1–3]. The approval of three CAR-T cell products by the US Food and Drug Administration (FDA), Yescarta, Kymriah and Tecartus, have paved the way for the clinical availability of CAR-T cell therapy [4]. CAR-T cell therapy is currently being tested in at least 600 clinical trials worldwide (www.clinicaltrials.gov).

Despite its success in patients with B-cell malignancies, there is a lack of substantive understanding of CAR-T cells in the human body. A typical multiphasic disposition profile of CAR-T cells consists of a rapid distribution phase leading to a time-restricted expansion phase, followed by contraction and prolonged persistence phases.

To date, there are no available standardized methods for monitoring in vivo behaviors of injected CAR-T cells. Although various imaging methods, such as radioisotope-labeled cells, genetically engineered cells (e.g., green fluorescent protein expression) and nanoparticle-labeled cells (e.g., iron-dextrannanoparticles), have been applied recently to characterize the distinct pharmacokinetic profiles of CAR-T cells [5], the most commonly used techniques such as flow cytometry, biopsy/immunohistochemistry (IHC), enzyme-linked immunosorbent (ELISpot) and polymerase chain reaction (PCR) cannot be discarded. Because most of the imaging methods can only monitor the CAR-T cells in a short time, common methods for long-time monitoring are needed.

Unlike conventional drugs, CAR-T cells act as a “living drug” that can proliferate in the body. They also exert functions for a significantly longer duration than conventional chemotherapeutics and antibody drugs [6]. Therefore, animal models are generally recommended for evaluating cell therapies because basic information of initial behavior, organ distribution and targeting in vivo after cell infusion are important.

To determine the distribution of CAR-T cells after administration, we conducted in vivo *assays* using NCG mice with or without tumor cells, and launched a small-scale clinical trial to study the pharmacokinetics of CD19 CAR-T cells in the blood of 13 B-NHL patients.

## Methods

### Cell culture and CAR-T cell product manufacture

CD19 CAR-T cells were designed for B-ALL and B-NHL by Beijing Immunochina Pharmaceuticals Co., Ltd. An FMC63-derived CD19-specific scFv, a CD8α-derived hinge and transmembrane domains, and a intracellular domain of CD3ζ with 4-1BB as the co-stimulatory signal domain constitute the CAR molecule. The process of building CAR has been described in the previous work [7]. Briefly, the PCR products of CAR molecules were ligated to the third-generation EF1α promoter-based lentiviral transfer plasmid pLenti6.3/V5 (Thermo Fisher, Waltham, MA, USA). The transfer plasmid, packaging plasmids (pLP1 and pLP2; Thermo Fisher), and envelope plasmid (pLP/VSVG; Thermo Fisher) were transfected into 293 T cells using polyethyleneimine (Polysciences, Warrington, PA, USA) to prepare the lentivirus. And then, 48 and 72 h after infection, the culture medium was collected, ultrafiltered and purified using Core 700 chromatography (GE Healthcare, Chicago, IL, USA).

The preparation of CAR T cells has been described in previous work [7]. Briefly, Peripheral blood mononuclear cells (PBMCs) were collected from volunteer’ (35 years old, male; for preclinical study) or patients’ (for clinical study) apheresis products, and prepared using Ficoll (GE Healthcare, Chicago, IL, USA). The T cells were isolated and activated using CD3/CD28 magnetic beads (Thermo Fisher). The X-VIVO 15 medium (Lonza Group, Basel, Switzerland) supplemented with 500 U/mL IL-2 was used for T cell culture. After 48 h, the cells were transfected with lentivirus at a multiplicity of infection (MOI) of 0.5. When CAR-T cells were cultured to sufficient numbers for testing or patient infusion, the cells were harvested. Then, the cells were suspended in cryopreserved solution at a density of 2 × 10^7^/mL and stored in a cell cryopreserved bag. Before transferring to liquid nitrogen for preservation, we use a programmed temperature drop apparatus to cool the cells.

NALM-6 (B-ALL cell line) purchased from ATCC in December 2016 (ATCC, Clone G5, CRL-3273™, 63943809), was cultured in RPMI 1640 containing fetal bovine serum (FBS; 10%, Wisent), L-glutamine (2 mmol/L, Gibco) and antibiotic-antimycotic (100×, Gibco). In the COA of cell line provided by ATCC, NALM-6 had been authenticated by STR analysis. Before being used in the experiment, the cells tested negative for mycoplasma.

Cell viability was determined by trypan blue staining with a staining time not more than 2 min.

### Biodistribution of CAR-T cells in NCG mice

Immunodeficient NCG (NOD/ShiLtJGpt-*Prkdc*^*em26Cd52*^*Il2rg*^*em26Cd22*^/Gpt) mice (6–8 weeks old) were purchased from GemPharmatech Co., Ltd. (Nanjing, China). All animal studies were approved by the Tsinghua University Animal Care and Use Committee (Beijing, China).

To detect the distribution of CAR-T cells without target cells, CD19 CAR-T cells (1 × 10^7^) in saline were intravenously injected into normal NCG mice, while mice treated with saline only served as controls. Three hours, D2, D8 and D15 after CAR-T infusion, thiopentone sodium was intraperitoneally injected into the mice for anesthesia. After anesthesia, blood was collected from the large vein behind the abdominal cavity with a volume of about 0.5 mL (anticoagulants), and the remaining blood cells were removed by heart perfusion. Heart, lungs, liver, kidneys, spleen, brain, stomach, duodenum, uterus, ovaries, testis, epididymis, bone marrow, adipose tissue and skeletal muscle were collected for CAR-T cell detection.

To establish a B-ALL model, the NCG mice were injected with 1 × 10^6^ NALM-6 cells via the tail vein. Five days later, the mice were intravenous injected with 5 × 10^6^ CD19 CAR-T cells in saline. Five minutes, 30 min, 1 h, 3 h, D1, D2, D7, D14, D28, D42 and D56 after CAR-T infusion, blood (anticoagulants) from six animals (3 male, 3 female) was acquired for CAR-T cell detection by qPCR and flow cytometry. At 3 h, D2, D7, D14, D28, D42 and D56 after CAR-T infusion, six animals (3 male, 3 female) were scarified every time and the organs were collected described above for CAR-T cell detection by qPCR.

### qPCR for CAR detection

For the qPCR assay to detect CAR-T cells, DNA from different tissues was extracted using a DNeasy Blood & Tissue Kit (Qiagen, 69,504) following the manufacturer’s instructions, and DNA concentrations were quantified using UV spectrophotometry and adjusted to a suitable concentration range. Primers and probes for CAR-T cells were designed and synthesized by Biomed Biotech (Beijing, China) as listed in Sup. Table 6. The PCR experimental conditions were: 95 °C for ten minutes, followed by 40 cycles of 95 °C for 5 s, 55 °C for 15 s and 72 °C for 35 s.

### Flow cytometry methods for CAR detection

To validate the CAR transduction efficacy and the changes of CAR T-cells in the blood after injection, we performed flow cytometry assay.

For CAR transduction efficacy, CAR-T cells (1 × 10^6^) were suspended in 100 μL Dulbecco’s PBS (DPBS; Thermo Fisher) and incubated with PE-conjugated anti-CD3 (BD, Biosciences) and FITC-conjugated anti-CAR (Immunochina Pharmaceuticals) for 30 min. After washing with DPBS twice, the cells were evaluated with FlowJo software (FlowJo 7.6.1).

For CAR-T cell detection, red blood cells were removed using an RBC lysing buffer (Sigma Aldrich, MO) for 5 min, followed by washing and re-suspension in 1× HBSS containing 1% FBS. The separated blood cells were stained with PE-conjugated anti-CD3 (BD, Biosciences) and FITC-conjugated anti-CAR (Immunochina Pharmaceuticals) in 4 °C for 30 min and followed by washing with DPBS containing 1% FBS. Cells were analyzed using BD FACS Callibur cytometry (BD Biosciences). The results were evaluated by FlowJo software.

### Clinical trial

An exploratory clinical trial (ClinicalTrials.gov Identifier: NCT03528421) was launched in r/r B-NHL patients who showed primary resistance or recurrence after at least two prior lines of systemic treatment, including anti-CD20 monoclonal antibody and anthracycline. The study was approved by Peking University Cancer Hospital, which was carried out from May 2018 to Nov 2019. Thirteen patients received fludarabine and cyclophosphamide for 3 consecutive days to deplete endogenous lymphocytes before CAR-T cell infusion. Response was evaluated based on the Lugano response evaluation criteria [8]. Peripheral CAR-T cell number, adverse events including cytokine release syndrome (CRS) and immune effector cell-associated neurotoxicity syndrome (ICANS), routine blood analysis, and blood biochemistry were monitored during follow-up study. CTCAE 5.0 (https://ctep.cancer.gov/protocolDevelopment/electronic_applications/ctc.htm) and ASTCT criteria [9] were utilized to grade the adverse events.

### Statistical analysis

All data represent mean ± standard deviation (SD) of n values, where n corresponds to the number of mice used. Analyses were performed using GraphPad Prism software (GraphPad Prism 8). The one-way ANOVA with pairwise comparison was performed to test the differences. A threshold of *P* < 0.05 was considered statistically significant for all analyses.

## Results

### CAR-T cells proliferated without target cells in NCG mice

**Fig. 1:**
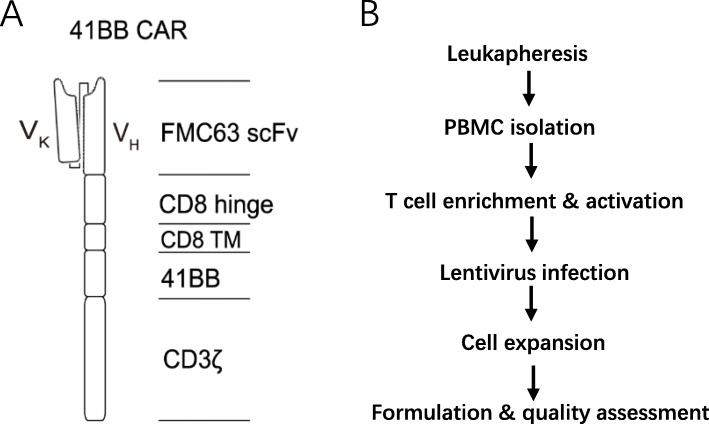
Basic information of CD19 CAR-T cells. **a**. The construction of CAR. FMC63-derived scFv with a 4-1BB co-stimulatory domain and a CD3ζ signaling domain. **b**. The manufacturing process of CAR-T products. CD3^+^ T cells were purified from PBMC and stimulated with CD3/CD28 Dynabeads. The T cells were then transfected with CAR lentivirus within 48 h and cultured for 9 days

CD19 CAR-T cells containing a 4-1BB co-stimulatory domain that can improve the expansion, persistence and antitumor effect of CAR-T cells [7, 10, 11] (Fig. 1a) were produced by Immunochina Pharmaceuticals following the described process (Fig. 1b).

**Fig. 2:**
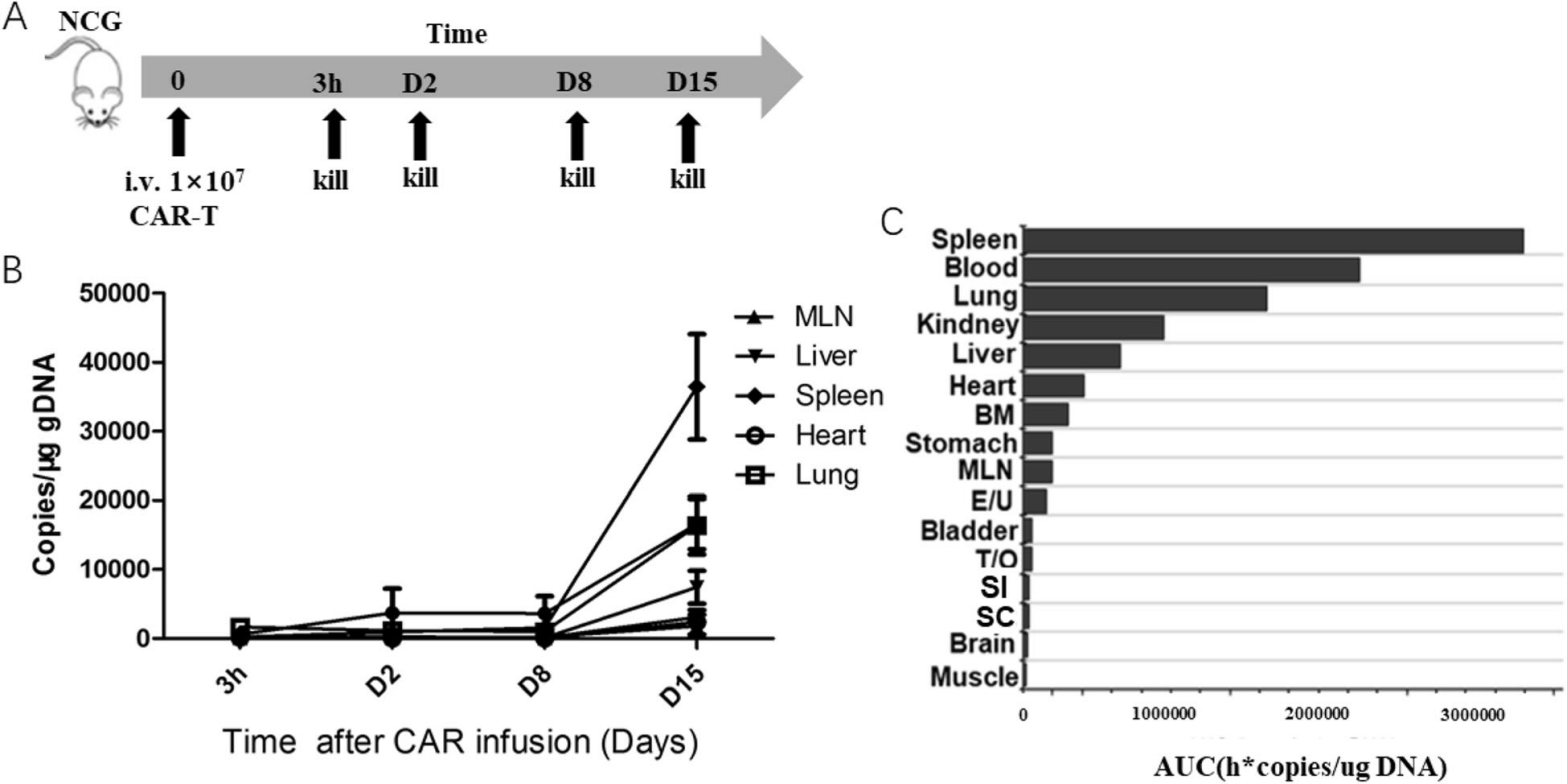
CAR-T distribution in the NCG mice. **a**. Flow diagram of the experiment. Mice were sacrificed at 3 h, day2, day8 and day15 after CAR-T infusion and the organs were collected to test the CAR gene copies. **b**. Changes of CAR-T cells in the organs over time (BM: bone marrow; MLN: Mesenteric Lymph nodes). **c**. Tissue exposure of CAR-T cells in NCG mice (AUC: Area under the curve; E/U: Epididymis/Uterus; T/O: Testis/Ovary; SI: Small Intestine; SC: Spinal Cord)

Most people believed that without target cells in mice, CAR-T cells will disappear in a short time after infusion. To verify this point, we produced CAR-T cells and transferred them into NCG mice through tail veins. One mouse was sacrificed at each set time point indicated in Fig. 2a and organs were collected for the CAR testing by qPCR. The results demonstrated that CAR-T cells still proliferated without target cells in mice. The CAR-T cell number increased markedly in every tissue especially in spleen within 2 weeks (Fig. 2b and Sup. Table 1). The area under the curve (AUC) showed that the spleen had the most CAR copies, which decreased significantly in the order of blood, lung, kidney, liver, heart and bone marrow. In the brain, muscle and reproductive organs, low CAR copies were detected (Fig. 2c and Sup. Table 2).

### CAR-T distribution in tumor-bearing mice

**Fig. 3:**
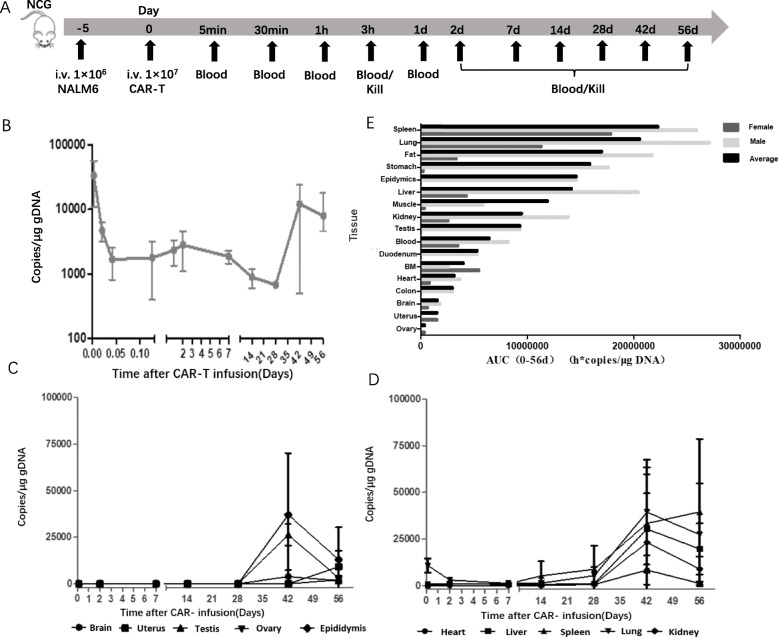
CAR-T distribution in the tumor-bearing mice. **a**. Flow diagram of the experiment. Blood and organs were taken from the tumor-bearing mice at different times (blood: 5 mins, 30 mins, 1 h and day1; blood and organs: 3 h, day2, day7, day14, day28, day42 and day56) after CAR-T cell infusion. **b**. Changes of CAR-T cell distribution in the blood over time. DNA from blood was extracted and the CAR gene copies were detected by qPCR. **c**. Changes of CAR-T cell distribution in the brain, uterus, testis, ovary and epididymis over time. DNA from 5 organs were extracted and the CAR gene copies were detected by qPCR. **d**. Changes of CAR-T cell distribution in the heart, liver, spleen, lung and kidney over time. DNA from 5 organs were extracted and the CAR gene copies were detected by qPCR. **e**. AUC of CAR-T cells in tissues and whole blood

To evaluate the distribution of CD19 CAR-T cells with target cells, we chose the NALM-6 tumor-bearing mice and infused CD19 CAR-T cells derived from healthy human donor into the mice. At different time points after CAR-T cell infusion, the whole blood and tissues were gathered for CAR-T cell testing (Fig. 3a).

Copies of CAR gene were detected in the peripheral blood of all animals 5 min after CAR-T cell infusion, which subsequently dropped to the lowest level at day 28 after treatment, then increased at day 42, and decreased again at day 56 (Fig. 3b and Table 1).

**Table 1:** Copies of CAR gene in blood at different time points (copies/μg DNA)

Time	Female		Male		Total	
	Mean	SD	Mean	SD	Mean	SD
5 min	45,135.5	29,994.5	22,675.7	6690.3	33,905.6	23,002.3
30 min	5223.4	1193.7	4219.6	1909.1	4721.5	1526.5
1 h	1615.1	889.4	1746.6	1045.9	1680.8	871.3
3 h	2000.6	1853.7	1446.2	620.9	1778.8	1380.8
1d	1664.2	410.8	2999.1	962.4	2331.7	986.2
2d	1787.9	1328.3	3852.3	1586.7	2820.1	1729.5
7d	1766.7	467.2	2023.3	466.4	1869.4	428.1
14d	746.2	410.1	1037.4	69.5	891.8	293.2
28d	711.6	/	641.3	/	676.5	49.7
42d	2132.1	522.0	18,925.6	10,244.5	12,208.2	11,711.1
56d	11,348.7	15,338.6	4543.3	4385.6	7946.0	10,013.6

Three hours after administration, CAR-T cells were mainly detected in the heart, liver and lung, and the content in the lung was the highest. CAR copies in spleen were detected in all animals 2 days after administration, followed by a slow increase to a peak at day 56. CAR-T cell detection in the other tissues, such as kidney, brain, stomach, duodenum, fat, muscle, colon, testis and epididymis, showed a decreasing trend during 2–14 days, and then gradually increased to the highest level at day 42. According to the results of CAR copy detection in various tissues, the number of CAR copies in most tissues increased at day 42 after administration, indicating that the activation and amplification of CAR-T cells appeared in most tissues (Fig. 3c/d and Sup. Table 3).

In general, the statistical data showed that CAR-T cells were mostly distributed in the spleen, followed by lung, fat, stomach, epididymis, liver, muscle, kidney, testis, blood, duodenum, bone marrow, heart and other tissues. The organ distribution of CAR-T cells in tumor-bearing mice was consistent with the distribution of cell products in vivo (Fig. 3e and Table 2).

**Table 2:** Summary of CAR-T parameters in tissues and whole blood (Mean)

	T_**max**_	Cmax	AUC_**0-1344h**_
Tissue	h	{copies/μg}	h*{copies/μg}
Heart	1008	8290.2	3,165,207.4
Liver	1008	30,327.7	14,164,836.0
Spleen	1344	39,484.9	22,287,294.0
Lung	1008	39,389.1	20,603,898.0
Kidney	1008	23,099.1	9,489,641.0
Brain	1008	3892.4	1,581,518.5
Uterus	1344	9159.5	1,538,796.4
Testis	1008	26,255.8	9,344,230.5
Ovary	1344	2157.9	362,522.2
Epididymis	1008	37,075.9	14,626,642.0
Stomach	1008	43,912.5	15,893,940.0
Duodenum	1008	15,141.3	5,315,737.1
Fat	1008	45,655.4	17,008,538.0
muscle	1008	34,794.5	11,934,449.0
Colon	1008	8374.4	2,996,989.2
Blood	0.083	33,905.6	6,448,893.4
Bone marrow	1344	8535.2	4,017,144.8

### CAR-T distribution and safety in NHL patients

Thirteen patients, 6 of whom were included in another paper to compare the effect of different co-stimulatory domains on the clinical outcomes of CD19 CAR-T cells [8], with diffuse large B cell lymphoma (DLBCL), follicular lymphoma (FL) or marginal zone lymphoma (MZL), were enrolled in this clinical trial. The characteristics of the patients are showed in Table 3.

**Table 3:** Patient characteristics and treatment

Patient No.	Age range	Diagnosis	Prior lines of treatment	Conditioning regimen before T cell infusion	CAR-T dosage
F0104	50–65	FL/II	> 2	Flu25mg/m^2^ + CTX250mg/m^2^	1 × 10^6^/kg
F0106	50–65	DLBCL	> 2	Flu25mg/m^2^ + CTX250mg/m^2^	1 × 10^6^/kg
F0107	< 50	FL/II	2	Flu25mg/m^2^ + CTX250mg/m^2^	1 × 10^6^/kg
F0109	50–65	DLBCL	> 2	Flu25mg/m^2^ + CTX250mg/m^2^	1 × 10^6^/kg
F0110	50–65	MZL	> 2	Flu25mg/m^2^ + CTX250mg/m^2^	1 × 10^6^/kg
F0111	< 50	DLBCL	> 2	Flu25mg/m^2^ + CTX250mg/m^2^	1 × 10^6^/kg
F0118	< 50	DLBCL	> 2	Flu25mg/m^2^ + CTX250mg/m^2^	1 × 10^6^/kg
F0119	50–65	DLBCL	> 2	Flu25mg/m^2^ + CTX250mg/m^2^	1 × 10^6^/kg
F0121	< 65	DLBCL	2	Flu25mg/m^2^ + CTX250mg/m^2^	1 × 10^6^/kg
F0122	50–65	DLBCL	2	Flu25mg/m^2^ + CTX250mg/m^2^	1 × 10^6^/kg
F0123	50–65	DLBCL	2	Flu25mg/m^2^ + CTX250mg/m^2^	1 × 10^6^/kg
F0125	50–65	DLBCL	2	Flu25mg/m^2^ + CTX250mg/m^2^	1 × 10^6^/kg
F0126	< 50	DLBCL	2	Flu25mg/m^2^ + CTX250mg/m^2^	1 × 10^6^/kg

Abbreviations: *MZL* marginal zone lymphoma, *DLBCL* diffuse large B cell lymphoma, *FL* follicular lymphoma, *Flu* fludarabine, *Cy* cyclophosphamide

**Fig. 4:**
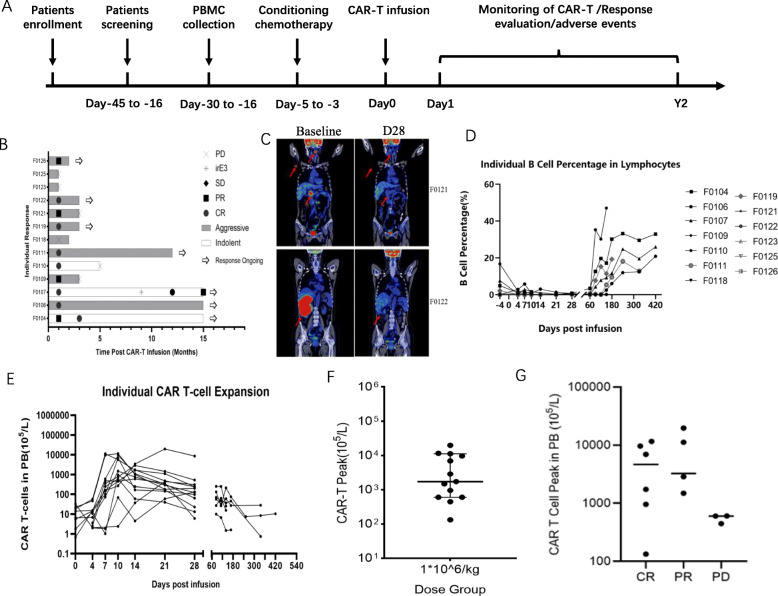
CAR-T distribution in NHL patients. **a**. Flow diagram of the experiment. After CAR-T cell infusion, 13 patients were followed by monitoring disease response, peripheral CAR-T cell number, adverse events including CRS and ICANS, routine blood analysis, and blood biochemistry. **b**. DOR of 13 patients. The first response was evaluated on day 28 and the longest monitoring duration was 15 months(DOR: Duration of Response). **c**. PET-CT data of Patient F0121 and F0122 at baseline and day 28 after CAR-T infusion. The tumors indicated by red arrows (Parameter: WW200 WL60 Fusion-70% SUVrange-0 to 6). **d**. The level of CD19^+^ cells in the peripheral blood of each patient after CAR-T cell therapy. **e**. Changes of CAR-T cells in peripheral blood after infusion in 13 patients. Red blood cells were removed followed by washing and re-suspension. The separated cells were stained with PE-conjugated anti-CD3 and FITC-conjugated anti-CAR. Data were acquired from the stained cells using BD FACS Callibur cytometry. The results were evaluated with FlowJo software. **f**. Average CAR-T cell peak in 13 patients. The average peak concentration of CAR-T cells was about 10^8^/L detected by FACS. **g**. The peak number of CAR-T cells in the peripheral blood of patients in CR, PR and PD groups

All patients underwent the preconditioning regimen to deplete endogenous lymphocytes before CAR-T cell infusion. After CAR-T cell infusion, the patients were followed by monitoring disease response, peripheral CAR-T cell number, and adverse events including CRS and ICANS, routine blood analysis, and blood biochemistry (Fig. 4a). The first response evaluation was on day 28, the complete remission (CR) rate was 46% (6/13), and 2 of the CR patients maintained remission for more than 15 months (Fig. 4b). PET-CT of Patient F0121 and F0122 showed that CD19 CAR-T cells could eliminate tumor cells effectively (Fig. 4c). We also monitored the level of CD19^+^ cells in the peripheral blood of each patient after CAR-T cell therapy, and found that B cell aplasia was induced by CAR-T cells (Fig. 4d). Expansion of CAR-T cells in peripheral blood was found in all patients, which reached the peak on day 7 to day 21. The persistence of CAR-T cells was detected up to 420 days (Fig. 4e and Sup. Table 4). The average peak concentration of CAR-T cells was about 10^8^/L (Fig. 4f and Sup. Table 5). Based on the clinical response of each patient on day 28 after CAR-T infusion, we divided the patients into three groups: CR, Partial Response (PR) group, and Progressive Disease (PD) group. The peak number of CAR-T cells in the peripheral blood of patients in each group was analyzed. We observed that the PD group showed relatively lower CAR-T cell peak value compared with the other two groups. However, the difference was not statistically significant (Fig. 4g).

About 53.8% (7/13) of the patients underwent grade 1 CRS and one of them developed to grade 2. The average duration of severe cytopenia events was 9.55 days, with a range from 2 to 32 days. The patients with severe cytopenia received recombinant human granulocyte colony-stimulating factor (G-CSF) as the only therapeutic intervention. The average duration of medication was 5.36 days, with a range from 1 to 12 days. All patients recovered from the adverse events after clinical intervention. None of the patients experienced ICANS [12]. Other side effects are summarized in Table 4. All the adverse events were effectively controlled within 2 months.

**Table 4:** List of other adverse events

Adverse events	Grade			
	I	II	III	IV
**Blood**				
Decrease of leukocyte count		3	5	3
Decrease of lymphocyte count		3		4
Decrease of neutrophil count		3	1	7
Decrease of platelet count	1	3	1	
Increase of C-reactive protein	2			
Degree III leukocyte bone marrow suppression			1	
Anemia	2	3	2	
Increase of serum ferritin	1			
**Liver**				
Elevated Alanine aminotransferase	1			
Elevated Aspartate aminotransferase	1			
**Pain**				
Pain in the jaws				1
Muscular soreness	1			
Pain in the left ilium		1		
**Immune globulin**				
Decrease of immunoglobulin A	3			
Decrease of immunoglobulin G	3			
Decrease of immunoglobulin M	3			
**Digestive tract**				
Diarrhea		2		
Gastrointestinal reaction	1			
Sick			1	
Vomit			1	
Dental ulcer	1			
**Respiratory tract**				
Cough	1			
Upper respiratory infection	1	1		
Pneumonia			3	
**Cardiovascular**				
Nodal tachycardia	2			
**Others**				
Insomnia		1		
Elevated urinary white blood cells		1		
Feeble	1			
Headache	1			
Elevated thyroid stimulating hormone	1			

## Discussion

CAR-T cell therapy is an effective new treatment for tumors [13–15] and three CAR-T cell products have been approved for clinical use by the US FDA [16]. But the distribution and location of the cells remain unclear in vivo. The influence of CAR-T cell peak in blood on the efficacy of CAR-T cell treatment also needs further investigation. Therefore, we conducted the preclinical and clinical study to investigate the distribution of CAR-T cells.

In our tumor-bearing mouse model, CAR-T cells were widely distributed in the organs well-perfused with blood, including the spleen, lung, fat, stomach, epididymis, liver, muscle, kidney, testis, duodenum, bone marrow and heart. They extensively spread to all over the organs from 4 weeks after administration and peaked between 6 and 7 weeks after administration. CAR-T cells also dramatically proliferated in NCG mice without target cells. One reason to explain the phenomenon might be that the T cell receptor (TCR) of CAR-T cells recognized xenogeneic Major Histocompatibility Complex I (MHCI) in mice and thus CAR-T cells were stimulated to proliferate. But this is only a hypothesis that requires experimental data to support its validity.

The process of T cell distribution is complicated, such as rolling and adhesion on vascular endothelial cells, chemokine-driven extravasation, and margination to specific tissues [17]. In different species, the process and characteristics of distribution could be diverse. So, we studied the CAR-T cell number in the blood of patients. Notably, in our study, the dynamic changes and peaks of CAR-T cells were not directly associated with the therapeutic efficacy, and the adverse events were inconsistent with the published literature [10, 18–20].

To summarize, we demonstrated that CAR-T cells can locate in different organs in mice, which indicated that CAR-T cells may also distribute in the tissues of humans. In this study, we only focused on understanding the quantitative changes of CAR-T cells in the blood of patients. Evaluation of the whole-body disposition of CAR-T cells in humans will be the next step to clarify the relationship between distribution and efficacy of CAR-T cells. To date, many new techniques have been developed to monitor the cellular location in human body, such as positron emission tomography (PET) [21, 22], bioluminescence imaging (BLI) [23] and so on. PET imaging of herpes simplex virus thymidine kinase 1 (HSV1-TK^+^) CAR-T cells co-expressing the CAR and the reporter gene of HSV1-TK within the same cell has been tested in patients with glioma [24]. We may detect the distribution of CD19 CAR-T cells in human body using one of these methods in the future.

Early translation of CAR-T cells in human must focus on safety and efficacy [25]. Some clinical studies about CAR-T cell therapy have indicated that severe and occasional fatal toxicities may occur [26–28]. CRS is the major toxicity [29]. ICANS is also emerging as a challenge for CAR-T cell therapies [30, 31]. Therefore, the prediction of side effects and efficacy is a significant project worth studying, and distribution research may lead to an important breakthrough.

## Conclusions

CAR-T cells can expand themselves with or without target cells in mice, and persist for a long time in NHL patients without serious side effects. The future direction is to explore the correlation between the expansion, distribution and clinical outcomes of patients treated with CD19 CAR-T cells.

## Supplementary Information

**Additional file 1: Table 1.** Distribution of CAR-T cells in NCG mice. **Table 2**. Tissue distribution parameters in NCG mice. **Table 3**. Distribution of CAR-T cells in tumor-bearing NCG mice. **Table 4**. Changes of CAR-T cells in the blood of patients over time. **Table 5**. CAR-T peak in the blood of patients during the therapy. **Table 6**. Sequences of primers and probes for CAR-T detection

## Data Availability

Please see the supplementary files.

## Abbreviations

AUC: Area Under The Curve
B-ALL: B-cell Acute Lymphoblastic Leukemia
B-NHL: B-cell Non-Hodgkin Lymphoma
BLI: Bioluminescence Imaging
CAR: Chimeric Antigen Receptor
CRS: Cytokine Release Syndrome
CR: Complete Remission
DLBCL: Diffuse Large B Cell Lymphoma
FDA: Food and Drug Administration
FL: Follicular Lymphoma
G-CSF: Granulocyte Colony-Stimulating Factor
IHC: Immunohistochemistry
ICANS: Immune Effector Cell-associated Neurotoxicity Syndrome
MHCI: Major Histocompatibility Complex I
MZL: Marginal Zone Lymphoma
PBMCs: Peripheral Blood Mononuclear Cells
PCR: Polymerase Chain Reaction
PD: Progressive Disease
PET: Positron Emission Tomography
PR: Partial Response
RCL: Replication-competent Lentivirus
TCR: T Cell Receptor
