# Supplementary material for: Distribution of chimeric antigen receptor-modified T cells against CD19 in B-cell malignancies
Source: BMC Cancer. 2021 Feb 25;21:198. doi: 10.1186/s12885-021-07934-1 (PMC7908740; doi:10.1186/s12885-021-07934-1)
Supplement: Supplementary file 1 — Additional file 1: Table 1. Distribution of CAR-T cells in NCG mice. Table 2. Tissue distribution parameters in NCG mice. Table 3. Distribution of CAR-T cells in tumor-bearing NCG mice. Table 4. Changes of CAR-T cells in the blood of patients over time. Table 5. CAR-T peak in the blood of patients during the therapy. Table 6. Sequences of primers and probes for CAR-T detection [file 12885_2021_7934_MOESM1_ESM.docx]

| Group | Time after CAR-T infustion | Gender | Number | Blood | Bone marrow | Mesenteric lympho node | Liver | Spleen | Heart | Lung |
| --- | --- | --- | --- | --- | --- | --- | --- | --- | --- | --- |
| Control  (PBS treated) | 3h | ♂ | 1716321 | BLQ | BLQ | BLQ | BLQ | BLQ | BLQ | BLQ* |
|  |  | ♀ | 1716324 | NA* | BLQ | BLQ | BLQ | BLQ | BLQ | BLQ |
|  |  |  | Mean | NA | NA | NA | NA | NA | NA | NA |
|  |  |  | SD | NA | NA | NA | NA | NA | NA | NA |
|  |  |  | CV% | NA | NA | NA | NA | NA | NA | NA |
| CAR-T | 3h | ♂ | 1716339 | BLQ* | 236.70* | BLQ | 77.32 | 382.27 | 110.72 | 2398.81 |
|  |  | ♀ | 1716342 | 1188.24* | BLQ* | BLQ | 179.97 | 83.55 | 99.50* | 961.08 |
|  |  |  | Mean | 594.12 | 118.35 | NA | 128.64 | 232.91 | 105.11 | 1679.95 |
|  |  |  | SD | 840.21 | 167.37 | NA | 72.59 | 211.23 | 7.93 | 1016.63 |
|  |  |  | CV% | 141.42 | 141.42 | NA | 56.43 | 90.69 | 7.55 | 60.52 |
|  | D2 | ♂ | 1716340 | 6198.71* | 369.17 | BLQ | 135.61 | 1112.85 | BLQ | 1360.60 |
|  |  | ♀ | 1716343 | 1218.41* | 193.47 | BLQ | 71.32 | 771.51 | BLQ | 938.98 |
|  |  |  | Mean | 3708.56 | 281.32 | NA | 103.47 | 942.18 | NA | 1149.79 |
|  |  |  | SD | 3521.60 | 124.24 | NA | 45.47 | 241.36 | NA | 298.13 |
|  |  |  | CV% | 94.96 | 44.16 | NA | 43.94 | 25.62 | NA | 25.93 |
|  | D8 | ♂ | 1716341 | 1874.98* | BLQ* | 97.34 | 107.91 | 1676.47 | BLQ | 1094.96 |
|  |  | ♀ | 1716344 | 5415.56* | 201.12* | 368.09 | 121.44 | 1477.80 | BLQ | 915.90 |
|  |  |  | Mean | 3645.27 | 100.56 | 232.71 | 114.67 | 1577.14 | NA | 1005.43 |
|  |  |  | SD | 2503.57 | 142.21 | 191.45 | 9.57 | 140.48 | NA | 126.61 |
|  |  |  | CV% | 68.68 | 141.42 | 82.27 | 8.34 | 8.91 | NA | 12.59 |
|  | D15 | ♂ | 1716314 | 14015.62* | 3295.15 | 3479.35 | 9802.36 | 28826.16 | 618.68 | 12190.47 |
|  |  | ♀ | 1716320 | 19163.12* | 2890.24 | 172.60 | 5079.62 | 44100.56 | 4135.33 | 20588.84 |
|  |  |  | Mean | 16589.37 | 3092.70 | 1825.98 | 7440.99 | 36463.36 | 2377.00 | 16389.65 |
|  |  |  | SD | 3639.83 | 286.32 | 2338.23 | 3339.48 | 10800.63 | 2486.65 | 5938.54 |
|  |  |  | CV% | 21.94 | 9.26 | 128.05 | 44.88 | 29.62 | 104.61 | 36.23 |

**Table 1. Distribution of CAR-T cells in NCG mice**

(BLQ indicates that the concentration is below the quantitative limit of the method; NA represents data that cannot be supplied or calculated; “*” means the sample nucleic acid extraction is not qualified**)**

| Tissue | N_Samples* | T_max_ | C_max_ | AUC_last_ |
| --- | --- | --- | --- | --- |
|  |  | h | {copies/μg} | h*{copies/μg} |
| Spleen | 4 | 336 | 36463.36 | 3389480.85 |
| Blood | 4 | 336 | 16589.37 | 2275254.84 |
| Lung | 4 | 336 | 16389.65 | 1648594.76 |
| Kidney | 1 | 336 | 5671.35 | 952786.80 |
| Liver | 4 | 336 | 7440.99 | 653011.64 |
| Heart | 2 | 336 | 2377.00 | 413428.98 |
| Bone marrow | 4 | 336 | 3092.70 | 300103.26 |
| Stomach | 1 | 336 | 1154.86 | 194016.48 |
| MLN | 2 | 336 | 1825.98 | 192477.60 |
| Epididymis/uterus | 1 | 336 | 937.16 | 157442.88 |
| Bladder | 1 | 336 | 326.31 | 54820.08 |
| Testis/ovary | 1 | 336 | 322.05 | 54104.40 |
| Intestine | 1 | 336 | 224.53 | 37721.04 |
| Spinal cord | 2 | 336 | 201.52 | 36749.28 |
| Brain | 2 | 336 | 108.03 | 25413.96 |
| Muscle | 1 | 336 | 94.23 | 15830.64 |

**Table 2. Tissue distribution parameters in NCG mice**

(MLN: Mesenteric lympho node)

| **Tissue** | **Gender** | **Type** | **Time after CAR-T infusion** | | | | |  |  |
| --- | --- | --- | --- | --- | --- | --- | --- | --- | --- |
|  |  |  | **3h** | **D2** | **D7** | **D14** | **D28** | **D42** | **D56** |
| Heart | F | Mean | 648.4 | BLQ | BLQ | BLQ | 515.9 | 1235.1 | 1410.8 |
|  |  | SD | 149.6 | / | / | / | / | / | 935.0 |
|  | M | Mean | 479.4 | BLQ | BLQ | BLQ | BLQ | 10641.9 | 874.7 |
|  |  | SD | 27.4 | / | / | / | / | 7652.7 | 616.0 |
|  | Average | Mean | 563.9 | BLQ | BLQ | BLQ | 515.9 | 8290.2 | 1142.7 |
|  |  | SD | 131.3 | / | / | / | / | 7820.8 | 716.7 |
| Liver | F | Mean | 817.6 | 1058.8 | 553.5 | BLQ | 1054.6 | 3659.4 | 15133.8 |
|  |  | SD | 178.6 | 217.0 | / | / | 215.1 | 1050.4 | 18770.4 |
|  | M | Mean | 555.9 | 834.3 | BLQ | 551.8 | BLQ | 48106.6 | 24160.6 |
|  |  | SD | 12.0 | / | / | / | / | 31585.0 | 11474.8 |
|  | Average | Mean | 712.9 | 1002.7 | 553.5 | 551.8 | 1054.6 | 30327.7 | 19647.2 |
|  |  | SD | 191.1 | 209.7 | / | / | 215.1 | 33041.6 | 13729.3 |
| Spleen | F | Mean | BLQ | 1063.6 | 1000.1 | 2022.7 | 12182.3 | 24862.3 | 27926.5 |
|  |  | SD | / | 399.1 | 457.4 | 1570.3 | 15765.7 | 15294.2 | 44789.6 |
|  | M | Mean | BLQ | 1000.5 | 725.0 | 9853.4 | 1719.1 | 39050.8 | 56822.5 |
|  |  | SD | / | 50.9 | / | 12685.1 | / | 33920.7 | 33534.1 |
|  | Average | Mean | BLQ | 1032.1 | 931.4 | 5155.0 | 8694.6 | 33375.4 | 39484.9 |
|  |  | SD | / | 256.8 | 398.0 | 7736.7 | 12679.6 | 26347.3 | 39175.0 |
| Lung | F | Mean | 13407.7 | 3789.1 | 1185.6 | 954.8 | 8811.6 | 11328.2 | 21045.2 |
|  |  | SD | 3520.0 | 682.7 | 196.6 | / | 3454.0 | 4754.4 | 33849.7 |
|  | M | Mean | 8002.2 | 2027.7 | 1132.3 | 1393.4 | 1793.6 | 58096.4 | 36752.5 |
|  |  | SD | 1698.8 | 753.2 | 312.0 | 914.6 | 1396.4 | 16242.4 | 21257.9 |
|  | Average | Mean | 10705.0 | 2908.4 | 1164.3 | 1247.2 | 5302.6 | 39389.1 | 27328.1 |
|  |  | SD | 3857.0 | 1159.4 | 211.0 | 694.6 | 4587.4 | 28173.4 | 27566.1 |
| Kidney | F | Mean | BLQ | BLQ | BLQ | BLQ | 744.8 | 2801.1 | 8202.6 |
|  |  | SD | / | / | / | / | 327.6 | 1777.0 | 10727.3 |
|  | M | Mean | BLQ | BLQ | BLQ | BLQ | BLQ | 36631.0 | 9393.8 |
|  |  | SD | / | / | / | / | / | 26547.2 | 4360.0 |
|  | Average | Mean | BLQ | BLQ | BLQ | BLQ | 744.8 | 23099.0 | 8798.2 |
|  |  | SD | / | / | / | / | 327.6 | 26391.4 | 6720.7 |
| Brain | F | Mean | BLQ | BLQ | BLQ | BLQ | BLQ | 884.8 | 2070.6 |
|  |  | SD | / | / | / | / | / | / | / |
|  | M | Mean | BLQ | BLQ | BLQ | BLQ | BLQ | 4894.9 | 1187.5 |
|  |  | SD | / | / | / | / | / | 3481.1 | / |
|  | Average | Mean | BLQ | BLQ | BLQ | BLQ | BLQ | 3892.4 | 1629.0 |
|  |  | SD | / | / | / | / | / | 3478.4 | 624.4 |
| Uterus | F | Mean | BLQ | BLQ | BLQ | BLQ | BLQ | BLQ | 9159.5 |
|  |  | SD | / | / | / | / | / | / | 8577.9 |
| Testis | M | Mean | BLQ | BLQ | BLQ | BLQ | BLQ | 26255.8 | 3108.8 |
|  |  | SD | / | / | / | / | / | 5735.2 | / |
| Ovary | F | Mean | BLQ | BLQ | BLQ | BLQ | BLQ | BLQ | 2157.9 |
|  |  | SD | / | / | / | / | / | / | 2125.9 |
| Epididymis | M | Mean | BLQ | BLQ | BLQ | BLQ | BLQ | 37075.9 | 12911.5 |
|  |  | SD | / | / | / | / | / | 33069.0 | 17463.9 |
| Stomach | F | Mean | BLQ | BLQ | BLQ | BLQ | BLQ | BLQ | 1507.2 |
|  |  | SD | / | / | / | / | / | / | 683.4 |
|  | M | Mean | BLQ | BLQ | BLQ | BLQ | BLQ | 43912.5 | 17330.9 |
|  |  | SD | / | / | / | / | / | 43710.2 | / |
|  | Average | Mean | BLQ | BLQ | BLQ | BLQ | BLQ | 43912.5 | 6781.8 |
|  |  | SD | / | / | / | / | / | 43710.2 | 9148.6 |
| Duodenum | F | Mean | BLQ | BLQ | BLQ | BLQ | BLQ | BLQ | BLQ |
|  |  | SD | / | / | / | / | / | / | / |
|  | M | Mean | BLQ | BLQ | BLQ | BLQ | BLQ | 15141.3 | 1358.7 |
|  |  | SD | / | / | / | / | / | 3373.6 | / |
|  | Average | Mean | BLQ | BLQ | BLQ | BLQ | BLQ | 15141.3 | 1358.7 |
|  |  | SD | / | / | / | / | / | 3373.6 | / |
| Fat | F | Mean | BLQ | BLQ | BLQ | BLQ | BLQ | 9133.5 | 1760.0 |
|  |  | SD | / | / | / | / | / | / | / |
|  | M | Mean | 628.5 | BLQ | BLQ | BLQ | BLQ | 57829.3 | 13872.7 |
|  |  | SD | / | / | / | / | / | 48839.8 | 13838.9 |
|  | Average | Mean | 628.5 | BLQ | BLQ | BLQ | BLQ | 45655.4 | 9835.2 |
|  |  | SD | / | / | / | / | / | 46723.0 | 12027.6 |
| Muscle | F | Mean | BLQ | 1252.0 | BLQ | BLQ | BLQ | BLQ | 1449.5 |
|  |  | SD | / | / | / | / | / | / | 239.6 |
|  | M | Mean | BLQ | BLQ | BLQ | BLQ | BLQ | 34794.5 | BLQ |
|  |  | SD | / | / | / | / | / | 32741.9 | / |
|  | Average | Mean | BLQ | BLQ | BLQ | BLQ | BLQ | 34794.5 | 1449.5 |
|  |  | SD | / | / | / | / | / | 32741.9 | 239.6 |
| Colon | F | Mean | BLQ | BLQ | BLQ | BLQ | BLQ | BLQ | BLQ |
|  |  | SD | / | / | / | / | / | / | / |
|  | M | Mean | BLQ | BLQ | BLQ | BLQ | BLQ | 8374.4 | 1090.4 |
|  |  | SD | / | / | / | / | / | 8381.9 | / |
|  | Average | Mean | BLQ | BLQ | BLQ | BLQ | BLQ | 8374.4 | 1090.4 |
|  |  | SD | / | / | / | / | / | 8381.9 | / |
| Bone  marrow | F | Mean | BLQ | 1906.2 | BLQ | BLQ | 1749.9 | 7173.7 | 13738.6 |
|  |  | SD | / | 1667.2 | / | / | 646.0 | 812.4 | 15588.9 |
|  | M | Mean | BLQ | 550.8 | BLQ | 453.0 | 1158.8 | 4538.4 | 3331.7 |
|  |  | SD | / | 194.8 | / | / | 649.3 | 3064.9 | 1084.4 |
|  | Average | Mean | BLQ | 1228.5 | BLQ | 453.0 | 1454.3 | 5592.5 | 8535.2 |
|  |  | SD | / | 1245.6 | / | / | 629.3 | 2635.4 | 10839.6 |

**Table 3. Distribution of CAR-T cells in tumor-bearing NCG mice**

(The above units were copies/ g DNA; "BLQ" means that the tested value of the sample is lower than the quantitative limit; "/" means incalculable.)

| **Patient NO.** | **Baseline** | **Time after CAR-T cell infusion** | | | | | | | | | |
| --- | --- | --- | --- | --- | --- | --- | --- | --- | --- | --- | --- |
|  |  | **D4** | **D7** | **D10** | **D14** | **D21** | **D28** | **M2** | **M3** | **M4** | **M5** |
| F0104 | 18.50 | 56.95 | 11170.18 | 5664.63 | 1666.62 | 1106.64 | 242.64 | 67.1 | 70.72 | 46.8 | 36.58 |
| F0106 | 22.62 | 46.62 | 9480.47 | 11554.95 | 1011.15 | 180.95 | 203.00 | 10.88 | 56.76 | 31.82 | 60.45 |
| F0107 | 1.76 | 13.33 | 102.18 | 6924.61 | 166.14 | 143.36 | 97.17 | 47.73 | 132.3 | 26.4 | 212.44 |
| F0109 | 14.07 | 15.20 | 152.50 | 270.60 | 3417.60 | 19656.00 | 8631.00 | 910.96 | 254.38 | 248.64 | 25.38(d135) |
| F0110 | 32.43 | 2.00 | 1.86 | 68.82 | 4.50 | 59.76 | 132.60 | 16.32 | 27.36 | 63.13 | / |
| F0111 | 0.00 | 11.00 | 1.03 | 956.92 | 248.24 | 166.14 | 22.95 | 1274.4 | 10.08 | 6.66 | / |
| F0118 | 0 | 2.48 | 251.34 | 600.48 | 59.59 | 39.78 | 13.23 | / | 4.71(d77) | / | / |
| F0119 | 5.52 | 14.4 | 597.4 | 9645.9 | 1098.9 | 332.52 | 64.8 | / | / | / | / |
| F0121 | 6.6 | 6.84 | 73.7 | 1483.93 | 753.03 | 292.05 | 123.07 | 15.84 | 9 | 4.05(d86） | / |
| F0122 | 0.68 | 0 | 159.46 | 459.13 | 1736.35 | 483.34 | 238.68 | 40.8 | 48.26 | / | / |
| F0123 | 0 | 2.05 | 0 | 2.4 | 37.17 | 446.49 | 309.6 | / | / | / | / |
| F0125 | 0 | 3.57 | 492.2 | 458.64 | 603.88 | 365.15 | 6.03 | / | / | / | / |
| F0126 | 0.00 | 0.00 | 356.90 | 767.52 | 2864.64 | 1443.03 | 528.84 | / | / | / | / |

**Table 4. Changes of CAR-T cells in the blood of patients over time**

(CAR-T cell number in blood,10^5/L; "/" means no detect)

| Patient NO. | Transfection efficiency | Cell viability | CD4:CD8 | Peak | Peak day |
| --- | --- | --- | --- | --- | --- |
| F0104 | 14.10% | 86.00% | 2 | 11170.18 | D7 |
| F0106 | 16.72% | 93.80% | 1.24 | 11554.95 | D10 |
| F0107 | 17.80% | 89.30% | 1.17 | 6924.61 | D10 |
| F0109 | 3.27% | 88.10% | 0.2 | 19656 | D21 |
| F0110 | 2.82% | 91.90% | 0.22 | 132.6 | D28 |
| F0111 | 2.36% | 91.00% | 0.06 | 956.92 | D10 |
| F0118 | 25.92% | 88.70% | 2.45 | 600.48 | D10 |
| F0119 | 23.86% | 88.70% | 0.58 | 9645.9 | D10 |
| F0121 | 46.09% | 91.90% | 0.64 | 1483.93 | D10 |
| F0122 | 42.88% | 91.70% | 0.83 | 1736.35 | D14 |
| F0123 | 22.48% | 86.60% | 0.38 | 446.49 | D21 |
| F0125 | 42.82% | 87.00% | 1.66 | 603.88 | D14 |
| F0126 | 37.56% | 88.20% | 1.69 | 2864.64 | D14 |

**Table 5. CAR-T peak in the blood of patients during the therapy**

(CAR-T cell number ,10^5/L)

| CAR-T Primers | Sequence(5’-3’) | Size(bp) |
| --- | --- | --- |
| WF Primer | GGCTTTCATTTTCTCCTCCTTGTA | 24 |
| WR Primer | CGGGCCACAACTCCTCATAA | 20 |
| Probe | FAM-ATCCTGGTTGCTGTCTC-MGBNFQ | 17 |

**Table 6. Sequences of primers and probes for CAR-T detection**
